# Unsupported gold nanocones as sonocatalytic agents with enhanced catalytic properties

**DOI:** 10.1016/j.ultsonch.2021.105753

**Published:** 2021-09-13

**Authors:** Xiaoqian Su, Umesh Sai Jonnalagadda, Lakshmi Deepika Bharatula, James Jing Kwan

**Affiliations:** a School of Chemical and Biomedical Engineering, Nanyang Technological University, 62 Nanyang Drive, 637459, Singapore; b Department of Engineering Sciences, University of Oxford, Oxford OX1 3PJ, United Kingdom

**Keywords:** Gold nanoparticles, Gold catalyst, Ultrasound, Cavitation, 4-nitrophenol

## Abstract

Gold catalysts have attracted attention for enabling sustainable chemical processes under ambient conditions. This reactivity is attributed to the small size of the catalysts (<5 nm); however, their size also creates difficulty when removing from product streams and often require rare-metal additives to enhance reaction rate kinetics, thereby limiting the environmental benefits of these catalysts. Comparatively, submicron gold catalysts are easier to separate but are much less reactive under ambient conditions. In this study, we synthesized submicron gas-stabilising gold nanocones (gs-AuNCs) that are acoustically responsive to afford greater reaction rates than other conventional gold catalysts. We explore the catalytic performance of acoustically responsive gs-AuNCs exposed to focussed ultrasound at 5.0 MPa peak negative pressure and 1.1 MHz center frequency. Cavitation nucleated from gs-AuNCs significantly increased the sonocatalytic degradation of water pollutants without the need for co-catalysts. The ability to amplify catalysis with ultrasound by tailoring the morphology of the catalyst to control cavitation opens new paths for future designs of sonocatalysts that may enable a sustainable chemical approach needed for a broad range of industrial processes.

## Highlights

• Submicron gas stabilising Au nanocones (gs-AuNCs) readily nucleate cavitation.
• Cavitation activates gs-AuNCs to degrade of 4-nitrophenol and methylene blue.
• Degradation kinetics with gs-AuNC are 200-fold faster than similarly sized catalysts.
• Cavitation events at catalyst sites enhanced functionality of submicron gs-AuNCs.

## 1 Introduction

Gold nanoparticles (AuNPs) have garnered interest due to their unique catalytic properties [1], [2], particularly their reactivity under ambient conditions for small particle clusters [3]. Present literature has demonstrated single Au atoms, bilayers, sub-nanometre clusters, clusters (1–2 nm), and nanoparticles (2–5 nm) as the active sizes of the Au species [4]. Unfortunately, because of their high surface energy, small Au catalysts are highly mobile and tend to aggregate during synthetic and catalytic processes. Therefore, fabrication of Au catalysts is often marred by complex doping methods to underlying support structures (e.g., TiO_2_, ZnO, etc) to enhance the native capabilities of the structure. These doping strategies are often accompanied by challenges with controlling the precise shape of the deposited gold clusters, rapid deactivation, and difficulties in catalyst recovery [4], [5]. Surface conversion of reactants may also be a rate-limiting step in the catalytic process since competition for catalytic surface sites affects the overall reaction rate [6]. Furthermore, the strong interactions between metal nanoparticles and the reducible oxides support may be an important mechanism for rapid catalyst deactivation leading to catalytic activity loss. Though unsupported larger (>100 nm in diameter) Au particles are more thermally and mechanically stable, and more amenable to production, filtration, and recovery, these particles still face the limitation of lacking the requisite catalytic properties under ambient conditions [7].

Recently, ultrasound has attracted attention as a sustainable stimulus to enable and enhance catalytic processing under ambient conditions [8]. The operating principle of ultrasound-mediated catalysis (sonocatalysis) is acoustic cavitation, a phenomenon that describes the oscillatory motion of a gas or vapour bubble in an acoustic field [9]. At low acoustic intensities, bubbles tend to undergo stable oscillations, resulting in local fluid streaming that can improve material dispersion. With larger acoustic intensities, the oscillations become more asymmetric and result in the uncontrolled expansion and eventual inertial collapse of the bubble, which is often referred to as inertial cavitation. This inertial collapse of the bubble generates localized physicochemical changes [10], which in turn may generate light (sonoluminescence) [11], free radicals [12], and local heating [13]. The local physicochemical changes from inertial cavitation accelerate chemical reactions under bulk ambient conditions. However, cavitation in a homogeneous fluid is a stochastic process; its inception requires immense acoustic energy, thereby making the process difficult to control and energetically costly for chemical processing [14]. To circumvent the high energy thresholds in homogenous fluids, the addition of exogenous gas nuclei, referred to as cavitation agents, reduces the energy required for cavitation and increases the rate of formation of cavitation bubbles [15], [16]. Cavitation agents (i.e., microbubbles) have been demonstrated to generate reactive oxygen species (ROS) through indirect activation of photosensitizers by cavitation induced sonoluminescence [17]. Recently, our group has taken this one step further by nanostructuring TiO_2_ particles to co-localize the catalytic site to the cavitation event for enhanced ROS formation [18].

Herein, we explore the potential of gas-stabilising gold nanocones (gs-AuNCs) to function as both cavitation nuclei and sonocatalyst, henceforth referred to as sonocatalytic cavitation agents. Unsupported gold nanocones (AuNCs) were fabricated with a well-defined submicron-scale conical “dendritic” structure with nano-scale multiple-branched petals. The surface cavities of AuNCs trap gas in the cavity. These surface-stabilised nanobubbles cavitate upon exposure to ultrasound. Acoustic noise from cavitation nucleated by gs-AuNCs exposed to focused ultrasound was measured with a passive cavitation detector (PCD). Additionally, we assessed the sonoreactivity of the gs-AuNCs in the presence of a reducing agent for degradation of 4-nitrophenol (4-NP) and methylene blue (MB), two model dyes conventionally used in gold catalysis and wastewater treatment [19], [20], [21], [22]. We also identified a direct correlation between the acoustic emissions generated by gs-AuNCs and the sonocatalytic degradation. Our results suggest that site-controlled cavitation plays a critical role in the efficient catalytic degradation of organic pollutants, guiding future advanced catalyst design for environmental remediation.

## 2 Materials and methods

### 2.1 Materials

Gold(III) chloride hydrate (HAuCl_4_, 99.999%), o-Phenetidine, hexane, sodium borohydride (NaBH_4_), 4-nitrophenol, and MB were purchased from Sigma-Aldrich and used as received. Agarose was bought from Vivantis Technologies. Deionized water was obtained from a pure water system (Stakpure, Germany).

### 2.2 AuNCs preparation

AuNCs were made using a method adapted from Zhang et al. [23]. Briefly, 5 mL of 0.8 mM HAuCl_4_ aqueous solution was preheated at 50℃ for 5 min. 2.5 mL of 20 mmol L^-^^1^ o-phenetidine in hexane was then gently layered on top of 0.8 mmol L^-^^1^ HAuCl_4_ aqueous solution. The mixture was transferred to a 9.5 L (29.21 cm × 24.1 cm × 15.2 cm) ultrasonic bath (Cole-Parmer 08895–83) to initiate an interfacial reaction at an operating frequency of 40 kHz and a power of 160 W. After continuous sonication at 50℃ for 30 min, the solution was cooled in an ice bath for one hour. The product was collected by centrifugation at 6000 rpm for 20 min. After freezing at -80℃ in an ultra-low-temperature freezer for 2 h, the samples were quickly transferred to a lyophilizer (Alpha 2–4 LSCbasic, Christ, Germany) and lyophilized for 24 h. After lyophilization, the samples were stored at -20℃ and sealed with parafilm to prevent moisture. Gas stabilization was accomplished by lyophilizing and resuspending the AuNCs so that they function as cavitation nuclei (gs-AuNCs).

### 2.3 AuNCs characterization

Size and morphology of AuNCs were obtained using a JEM-1400 (JEOL, Japan) transmission electron microscopy (TEM). Samples for TEM imaging were prepared by adding 10 μL of aqueous dispersions on 300-mesh carbon-coated copper grids. The grids were air-dried at room temperature. Size distributions were determined by dynamic light scattering (DLS) (Malvern Nano-ZS). The localised surface plasmon resonance peak of GNCs was detected using a UV–vis Spectrometer (Shimadzu UV 2450). The crystal structure of the AuNCs was examined by X-ray diffraction (XRD, Bruker D2 Phaser) by Cu Kα radiation with an accelerating voltage and current at 30 kV and 10 mA, respectively. The phase angle was adjusted between 5° and 40° at 0.05° increments (2Θ = 10–80°) with a scan time of 0.5 s at each step).

### 2.4 Acoustic response from AuNCs

A conventional high intensity focused ultrasound (HIFU) setup was used in all HIFU experiments (Figure S1a) [18], [24], [25]. To assess the cavitation potential of the particles through a range of pressures, an acoustically transparent flow chamber was constructed (Figure S1b) and aligned at the focus of high intensity focused ultrasound transducer (1.1 MHz, Sonic Concept H102). gs-AuNCs were prepared at a 1, 0.1, 0.05, and 0.01 mg/mL working concentration in aqueous media and loaded into the continuous flow chamber at a continuous and constant flow of 200 μL/min through the channel for acoustic excitation. The ultrasound transducer was driven by a function generator (Keysight 33210A) and an RF power amplifier (Electronics & Innovation 1040L). Particles were exposed to 20 cycle bursts for 20 s with increasing peak negative pressure amplitude (0.2–5.2 MPa peak negative pressure) at a pulse repetition time of 0.1 s. Acoustic emissions from particles were detected using a 15 MHz PCD (15 MHz, Olympus, Japan VU-V319) co-axially aligned with the transducer. The PCD signal was filtered utilizing an analogue 2.5 MHz high-pass filter (Allen Avionics F5286-2P50-B) before amplification through a broadband amplifier (5x, SRS SR445A). This processed signal was captured on the oscilloscope (National Instruments, USA PCI-5122) and saved for later processing as described previously [24]. The recorded signal was post-processed by a power fast Fourier transform (FFT) to determine the power spectral density (PSD) curve [26]. For each burst, the area under the PSD curve was determined and compared to degassed water exposed to ultrasound under the same conditions. Following the signal processing, cavitation was said to occur if the received signals were 6 dB higher than noise from the water control. The probability of cavitation at each pressure level was determined as the ratio of bursts that recorded a cavitation event out of the total number of ultrasound bursts ($p\left(P\right)=\frac{P_{pulses,cavitation}}{P_{pulses,total}}$). Acoustic amplitudes in this study are reported in MPa peak negative pressures. To determine the PCD signal “energy” from the measured acoustic noise (henceforth referred to as cavitation energy) emitted from the sonochemical reactor during ultrasound exposure, we summed the area under the PSD curve of each burst.

### 2.5 Catalytic study on 4-nitrophenol

The catalytic performance of gs-AuNCs was validated in an acoustically transparent static flow chamber (Figure S1c). 4-NP (0.5 mL, 0.1 mmol L^-^^1^) was mixed with fresh NaBH_4_ solution (0.5 mL, 0.5 mmol L^-^^1^). Then 50 μL of 2 mg/mL of gs-AuNCs resuspensions were added into the solutions. The reaction mixture was transferred into the static chamber for high intensity focused ultrasound exposure (1.1 MHz, 5 MPa peak negative pressure, and 50% duty cycle). After irradiation, the solutions were collected from the reaction chamber and centrifuged at 9000 RCF for 5 min to pellet the nanoparticles. The rate of catalytic reaction was determined using UV–vis spectroscopy.

### 2.6 Catalytic study on MB

For MB degradation, 0.5 mL of 0.03 mmol L^-^^1^ MB solution was mixed with 0.5 mL of 0.05 mmol L*^-^*^1^ fresh NaBH_4_ solution. Then 50 μL of 2 mg/mL of gs-AuNCs resuspensions were added into the solutions. The reaction mixture was transferred into the static chamber for HIFU exposure (1.1 MHz, 5 MPa peak negative pressure, and 20% duty cycle). After irradiation, the solutions were collected from the reaction chamber and centrifuged at 9000 RCF for 5 min to pellet the nanoparticles. The rate of catalytic reaction was determined using UV–vis spectroscopy.

The first order rate kinetics were calculated using linear regression on the concentration change with time according to the equation $-ln\left(\frac{C_{i}}{C_{o}}\right)=kt$, where $C_{i}$ is the concentration of dye at a given ultrasound exposure period, C_0_ is the initial dye concentration at t = 0 min, and *k* is the first order rate constant. Then the total acoustic emissions relevant to inertial cavitation (cavitation energy) was related to the %Degradation of each dye.

The catalytic stability and reusability of the gs-AuNCs sonocatalytic cavitation agents has been evaluated. The catalytic activity measurements were performed for 6 cycles under the same reaction conditions. After each cycle, the catalysts were separated from the reaction mixture by centrifugation, washed with deionized water, freeze-dried, and reused for the next cycle.

## 3 Results and discussion

### 3.1 Synthesis and characterization of AuNCs

**Figure 1:**
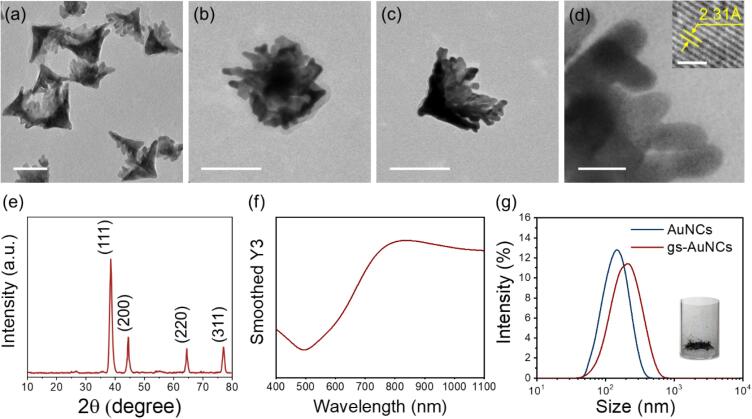
Physical characterization of AuNCs. TEM (a-d) and high resolution TEM (inset of d) images, bottom view (b), side view (c), and edges (d) of AuNCs. (e) XRD pattern of AuNCs. (f) UV-vis absorbance spectra of AuNCs. (g) Size measurements of AuNCs before and after lyophilization. The inset shows a photograph of lyophilized AuNCs. Scale bars present 100 nm in (a–c), 10 nm in (d), and 1 nm in (inset of d).

AuNCs were synthesized by following the ultrasound-assisted interfacial growth method [23]. In brief, upon ultrasound sonication, Au nuclei are immediately produced, and nuclei rapidly grow into hemispherical shells at the oil–water interface by a reducing HAuCl_4_ with o-phenetidine. Upon the continuous sonication, ultrasound results in the formation (vaporization of oil phase) and inertial collapse of bubbles, leading to the morphological transition of the half shells to conical structures*.* As shown in Fig. 1a, the obtained cone-shaped particles present relatively sharp tips and broad opening bottoms with jagged edges. The base diameters of AuNCs were measured to be 169.5 ± 21.70 nm and the cone height was a length of 115.9 ± 17.1 nm. Fig. 1b and c show bottom and side TEM images of AuNCs. The AuNCs appear to have well-defined hollow cavities comprising of “dendritic” structures, consisting of branching petals (∼6.8 nm) and narrow gaps (1–2 nm, Fig. 1d). The high-resolution TEM image (inset of Fig. 1d) indicates that these petals are single crystals that grew along with the (1 1 1) facets with a d-spacing of 0.23 nm. The crystallinity of AuNCs was further investigated using XRD (Fig. 1e), where AuNCs exhibited 4 characteristic diffraction peaks matched with (1 1 1), (2 0 0), (2 2 0), and (3 1 1) crystal planes of face-centred cubic gold. AuNCs also exhibited a characteristic localized surface plasmon resonance (SPR) peak at 830 nm (Fig. 1f). Gas stabilization on the AuNCs (gs-AuNCs) was successfully accomplished by drying the AuNCs and re-suspending the powder in degassed deionized water. Subsequent particle size analysis showed the hydrodynamic diameters to be 142.1 ± 13.9 nm for AuNCs and 205.0 ± 32.9 nm for gs-AuNCs (Fig. 1g). This size increase is suspected to be a result of the surface stabilized bubble, rather than particle aggregation [15].

### 3.2 Acoustic response of AuNCs

**Figure 2:**
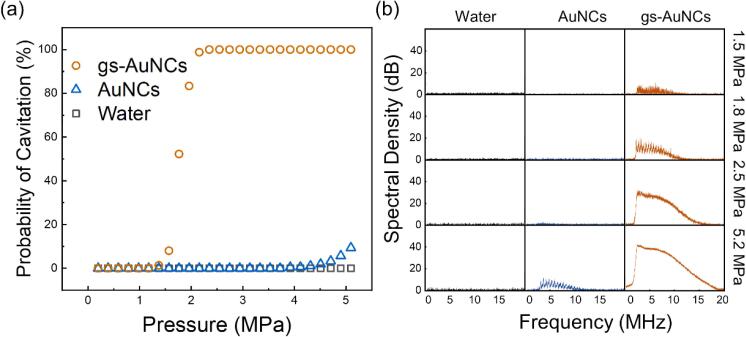
Cavitation potential of AuNCs at different pressures. (a) Probability of cavitation of DI water, AuNCs, and gs-AuNCs exposed to 20 cycle bursts of 1.1 MHz ultrasound for 20 s with increasing peak negative pressure amplitude from 0.2-5.2 MPa peak negative pressure at a pulse repetition time of 0.1 s. (b) The normalized spectral density curves for DI water, AuNCs, and gs-AuNCs below cavitation threshold (1.5 MPa), at cavitation threshold (1.8 MPa), above cavitation threshold (2.5 MPa), and at maximum tested pressure (5.2 MPa).

The acoustic response of AuNCs was assessed using a conventional high intensity focused ultrasound (HIFU) setup (Figure S1) at 1.1 MHz with 20 cycle bursts at a pressure ramp from 0.2 to 5.2 MPa [24], [27]. Voltage traces from the PCD were post-processed to determine the presence of cavitation and relative intensity of the cavitation noise. From these measurements, we calculated the probability for inertial cavitation and determined cavitation threshold (pressure at 50% cavitation) for 1 mg/mL of gs-AuNCs (Fig. 2a). gs-AuNCs displayed cavitation at peak negative pressure amplitudes of at least 1.3 MPa with the likelihood of cavitation monotonically increasing to 100% at 2.5 MPa. The cavitation threshold was found to be at 1.8 MPa. Comparatively, the lack of bubbles trapped on AuNCs reduced the acoustic response of the particles to 4.5 MPa. These AuNCs demonstrated infrequent cavitation (probability < 10%) at the maximum tested peak negative pressure amplitude of 5.2 MPa, likely due to the AuNCs functioning as a local defect to the cavitation threshold of pure water. Interestingly, deionized water did not cavitate at any pressure amplitude tested emphasizing the importance of gas trapping on the nanocones. Comparatively, gs-AuNCs significantly reduced the cavitation threshold due to the gas trapped (within the cavity or on the surface) during the drying and resuspension process [28]. In the context of sonochemistry, inducing cavitation events in the homogeneous fluid media require greater acoustic intensities, higher duty cycles, and longer irradiation times than the intensities assessed here [29], [30].

Interestingly, the cavitation threshold of gs-AuNCs was substantially lower than other similarly sized nanoparticles [31]. This may be attributed to the geometry of the AuNCs [32]. Because of the conical shape of the AuNCs and relatively thin walls, most of the AuNCs are comprised of empty space. This empty space is able to accommodate a larger volume of gas compared to both mesoporous and shell-stabilized bubble systems [24], [33]. Also, the trapped nanobubbles on the gs-AuNCs are easily excitable due to the wide-opening of cone-shaped gold nanoparticles, thereby substantially reducing the inertial cavitation threshold. By investigating the frequency content of the acoustic response, it was evident that there was a distinct change in intensity and composition of the received signal with increasing pressure (Fig. 2b). We observed that AuNCs exhibited acoustic emissions at 5.2 MPa, which was in agreement with our earlier observations presented in Fig. 2a. gs-AuNCs generated prominent harmonic noise at pressure amplitudes of 1.5 MPa (below cavitation threshold), while generated both harmonic and broadband signals at 1.8 MPa (at threshold). Above 2.5 MPa (at 100% cavitation), gs-AuNCs generated broadband dominant signals. This behaviour is comparable with other sub-micron systems over similar acoustic parameters [15]. We further validated the acoustic response of the gs-AuNCs at a range of concentrations of 0.01, 0.05, and 0.1 mg/mL (Figure S2). At a lower concentration of 0.01 mg/mL, the cavitation was found unreliable (<5%). Comparatively, the gs-AuNCs at 0.05 mg/mL reached 50% of cavitation at 5.0 MPa and the cavitation threshold further reduced to 3.1 MPa peak negative pressure when increasing the concentration to 0.1 mg/mL. Given this, it is clear that cavitation response is proportional to the concentration of gs-AuNCs. In our study, we used 0.1 mg/mL gs-AuNCs as a baseline concentration and utilized a peak negative pressure of 5 MPa to ensure 100% cavitation throughout the irradiation period for all subsequent tests.

### 3.3 Sonocatalytic degradation of 4-NP and MB with gs-AuNCs

We next validated the sonoreactivity of the particles using two established dyes (4-NP and MB) found in water treatment literature [19], [20]. Current gold-based catalysts have shown great potential in degradation of 4-NP and similar organic pollutants [21], [22]. Typically, these reactions occur in the presence of supporting materials (e.g. metal oxides, carbon, organic frameworks) and reducing agents (e.g. NaBH_4_) to enhance electron transfer [19], [21], [22]. Here, we show cavitation and its aforementioned effects co-localised with the catalytic surface will improve the catalytic reaction rates of Au under ambient conditions. Inertial cavitation from our gs-AuNCs serves two purposes. First, bubble collapse nucleated from gs-AuNCs directly converts water into hydroxyl radicals, hydrogen peroxide, and other ROS via pyrolysis. Second, thermal and mechanical effects from a collapsing bubble may improve the catalytic performance of gs-AuNCs.

**Figure 3:**
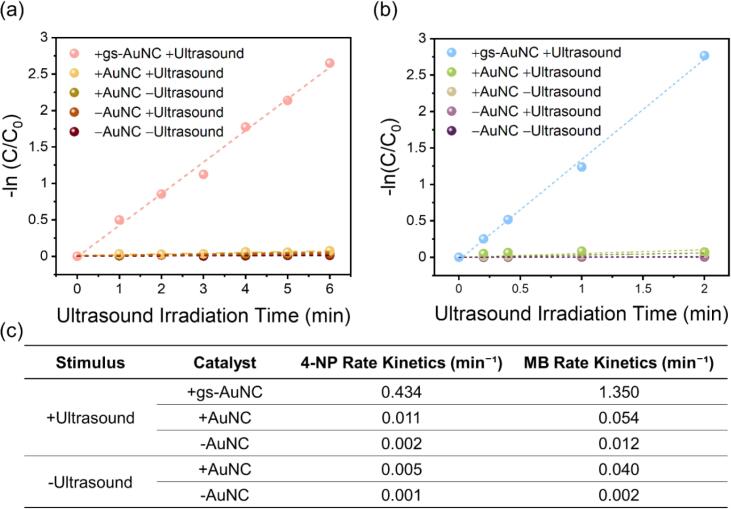
Sonoreactivity of gs-AuNCs for degradation of model dyes under 1.1 MHz ultrasound irradiation at 5.0 MPa peak negative pressure. Sonocatalytic degradation presented for 4-NP and MB with gs-AuNCs, AuNCs, or no catalyst. The quantified first order rate kinetics for 4-NP (a) and MB (b) have been calculated at these different conditions and presented as a function of ultrasound irradiation time. A tabulated summary of the rate kinetics for all conditions is given in (c).

We set out to demonstrate gs-AuNC-based sonocatalysis on 4-NP and MB degradation as model reactions. We utilized UV–vis to confirm that chemical conversion of 4-NP (Figure S3) and MB (Figure S4) occurs with ultrasound irradiation in the presence of gs-AuNCs. Given that we utilized pulsed ultrasound, we defined ultrasound irradiation time as the total time the ultrasound transducer was active. Here, the ultrasound irradiation time was 20–50% of the total exposure time, i.e., the duty cycle was 20–50%. We found that degradation of 4-NP and MB was exhibited within 6 min and 2 min of ultrasound irradiation time, respectively. To validate that this enhancement was due to site-specific cavitation from gs-AuNCs, 4-NP and MB solutions with non-gas stabilised AuNCs were exposed to ultrasound under the same acoustic parameters. Without trapped gas, AuNCs exhibited fewer cavitation events during ultrasound irradiation (Fig. 2b) and subsequently resulted in less degradation of the dyes over the same time points for both 4-NP and MB, which are generally more similar to the AuNCs without ultrasound irritation (Fig.  3 and S5). In the absence of AuNCs, the degradation of 4-NP and MB solution proceeded very slowly with or without ultrasound irradiation. The rate kinetics for all conditions are summarized in Fig.  3c. In brief, ultrasonic irradiation of the solutions in the presence of gs-AuNCs resulted in an exponential degradation for both dyes whereby the degradation rates of 4-NP and MB increased by 87-fold to 0.43 min^-^^1^ and 34-fold to 1.35 min^-^^1^ compared to the no ultrasound group (0.005 and 0.04, respectively). These measurements suggested that localized cavitation events onto the catalyst played a vital role in the reaction.

**Figure 4:**
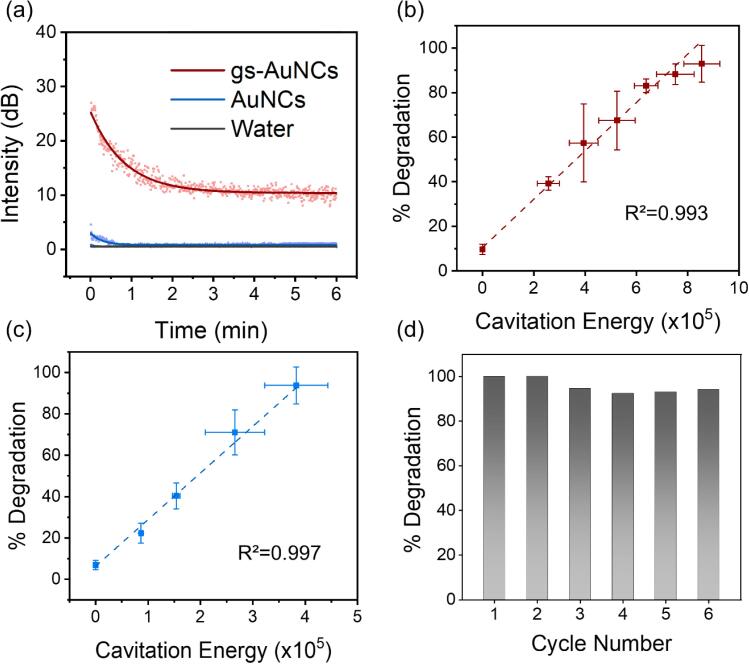
gs-AuNCs cavitation dynamics correlation to sonocatalytic reaction kinetics and the recyclability of gs-AuNCs. (a) Acoustic intensities change over time during sonochemical reactions. Sonocatalytic degradation of 4-NP (b) and MB (c) correlated to total received cavitation energy. Data represented as mean ± SD (n=3). (d) The recycling stability of gs-AuNCs for catalytic degradation of MB.

Though cavitation has previously been studied for chemical processing [30], [34], [35], the intensity of inertial cavitation during sonochemistry is not often quantified. Here, we evaluated the correlation between the percentage of sonodegradation of the dyes at different ultrasound irradiation durations to the measured PCD signal energy, which is proportional to the cavitation energy across that duration. When normalizing this energy against a reference signal, we can further observe acoustic intensity of sonochemical reactor change over time (Fig. 4a). The results demonstrated gs-AuNCs sustained cavitation activity throughout the 6 min exposure with a decay at the beginning. Similar to the behaviour presented in Fig. 2a, AuNCs presented virtually identical cavitation behaviour as water, further demonstrating that the presence of cavitation was due to the gas trapped by the gs-AuNCs. The recorded PCD signal energy proportional to inertial cavitation (referred to as cavitation energy here) from gs-AuNCs throughout the reaction were correlated to 4-NP and MB degradation (Fig. 4b and c). We observed a direct positive correlation between the cavitation response of the sonochemical reactor and degradation for both 4-NP (Fig. 4b) and MB (Fig. 4c). Given our findings in Fig. 3, whereby gs-AuNCs with ultrasound irradiation demonstrated an exponential change in 4-NP and MB degradation, it is evident that catalyst site-specific cavitation was critical for gs-AuNC-mediated sonocatalysis. Interestingly, less cavitation energy was required to fully degrade MB compared to 4-nitrophenol, leading to a more exponential change in degradation with cavitation energy.

The submicron gs-AuNCs sonocatalytic cavitation agents efficiently sonocatalyticaly degraded 4-NP and MB. Because of the submicron-scale of the gs-AuNCs, they can be easily separated through centrifugation, dried and resuspended to re-trap gas, and recycled for future reactions. Fig. 4d shows the recycling efficacy of gs-AuNCs by comparing the sonocatalytic degradation of MB across six cycles. Here, it can be observed that the percent degradation of MB was consistently >90% across the six cycles of reaction, demonstrating the persistent stability, sonoreactivity, and reusability of gs-AuNCs in the presence of ultrasound.

The effect of the dosage of NaBH_4_ and gs-AuNCs on the catalytic degradation of MB have also been evaluated. Figure S6 shows the effect of NaBH_4_ concentration on the sonodegradation of MB by gs-AuNCs. The reaction rates of the sonocatalytic degradation of MB were significantly enhanced by increasing the dosage of NaBH_4,_ and nearly complete degradation of MB was observed with 2 min of ultrasound irradiation at 0.05 and 0.1 mmol L^-^^1^. The rate for MB degradation increases from 0.09 min^-^^1^ at 0 mmol L^-^^1^ NaBH_4_ to 1.35 at 0.05 mmol L^-^^1^ NaBH_4_ and then levels off to a plateau of 1.6 min^-^^1^ at 0.1 mmol L^-^^1^ NaBH_4_. Figure S7 shows the effect of gs-AuNCs concentration on the degradation efficiency of MB. The rate increases significantly from 0.16 min^-^^1^ at 0.05 mg/mL gs-AuNCs to 4.66 min^-^^1^ at 1 mg/mL gs-AuNCs. The increase in rate for MB degradation upon increasing concentration of gs-AuNCs is due to the increased reactive sites of Au and higher inertial cavitation generation (which agreed with our earlier observations presented in Figure S2) during the sonochemical reactions.

The catalytic activity of gold decreases with increasing particle size [7]. In this study, submicron non-supported gs-AuNCs with a dendritic structure was reported as both cavitation nuclei and catalyst for fast organic pollutant degradation. gs-AuNCs present a comparable rate kinetic to existing Au catalysts with much smaller sizes under similar reaction conditions [7], [36]. This enhanced catalytic activity for submicron gs-AuNCs may be attributed to the unique shape of the nanocones. By nanostructuring the gold nanocones with nano-branched petals with long narrow gaps (1–2 nm) the catalytic reactivity may be enhanced and, most importantly, gs-AuNCs were able to nucleate inertial cavitation around the catalytic site during ultrasound irradiation. To evaluate the efficacy of our sonocatalytic approach, we compared our reaction rates to those presented in the literature for 4-NP degradation using gold or gold composite nanoparticles (Table 1). Our sonocatalytic approach to using the catalyst as the cavitation nuclei enhanced the catalytic potential of gold nanoparticles at rates of 62-fold higher than 10 nm unsupported Au catalysts [36], 200-fold faster than 55 nm and 27-fold higher than 8 nm resin beads supported spherical Au catalysts [7], and 9-fold higher than 14 nm supported heterogeneous Au catalysts [37]. The reason for this enhanced catalytic capability of gs-AuNCs was attributed to nanostructuring of AuNCs combined with the sonoactivation. Sonoactivation of gs-AuNCs allows for more effective Au catalysis by colocalizing cavitation events at catalytic sites. By similar metrics, we found that our system demonstrates more rapid MB degradation than much of the existing literature (Table S1).

**Table 1:** Comparison of catalytic activity of Au by degradation of 4-NP.

Catalysts	Au Size (nm)	Dose (mg·mL^-^^1^)	*k* (min^-^^1^)	*K*^a^ (min^-^^1^ mg^-^^1^)	Ref
Au + Ultrasound^b^	170	0.1	0.434	4.340	This work
Au	170	0.1	0.005	0.050	This work
Au	10	–	0.007	–	[36]
Au/Resin	8	2	0.016	0.008	[7]
Au/Resin	55	2	0.002	0.001	[7]
Fe_3_O_4_@COF-Au	4	0.27	0.222	0.822	[19]
Au@Ag@PDA/rGO	14	–	0.050	–	[37]
p(AAm-co-TMT)@Au	10–20	0.05	0.136	2.720	[38]
Au/CeO_2_/rGO	3	1	0.438	0.438	[39]
Au/OMS	4	0.1	0.058	0.58	[40]

a Normalised rate constant *K* is the ratio of the apparent rate constant (*k*) to the mass of catalyst (mg).

b 1.1 MHz focussed ultrasound irradiation at 5.0 MPa peak negative pressure and 50% duty cycle.

### 3.4 Proposed mechanism for gs-AuNC derived sonocatalysis

In this study, we demonstrate how co-localisation of the cavitation events onto the catalyst dramatically improves the catalytic performance of gs-AuNCs. This faster catalytic sonochemistry may be due to thermal, mechanical, and chemical effects of cavitation nucleated from gas trapped in AuNCs [13]. By stabilizing gas onto the Au cavities, cavitation events more readily nucleate at the catalytic site; the bubble is the primary defect that enables cavitation and it has been shown that cavitation from gas-stabilising solids occurs at the nanoparticle [41]. As a result, there will exist a temporary and localised high-energy microenvironment primed for catalytic reactions under bulk ambient conditions.

**Scheme 1:**
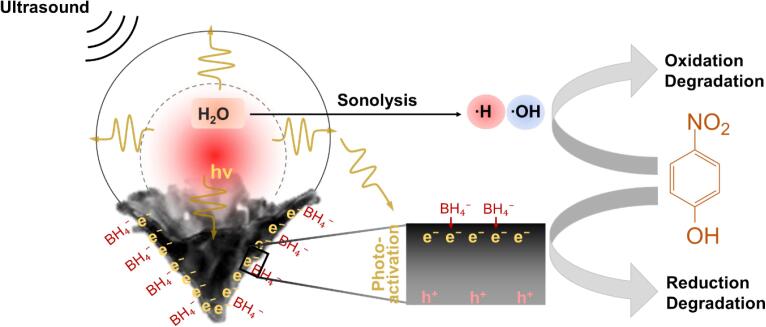
Schematic illustration of gas trapping by AuNCs and generation of cavitation event by ultrasound exposure (1.1 MHz focussed ultrasound at 5 MPa peak negative pressure). Upon bubble collapse, hot pot generated and sonolysis of water incurs to generate hydrogen and hydroxyl radicals (⋅H and ⋅OH, respectively). Sonoluminescence is also generated by the cavitation event, which will enhance electron transfer along the gold surface synergistically with the borohydride ions for more efficient reduction kinetics.

Within this cavitation-induced high-energy microenvironment, there is a multitude of physical and chemical effects occurring in parallel that may enhance catalysis (Scheme 1). For 4-NP and MB reactions, the reactant ions and a hydrogen species derived from BH_4_^-^ first adsorb onto the surface of the catalyst. A reduction reaction then occurs via electron transfer from the donor BH_4_^-^ to the acceptor reactants on the surface of Au catalyst. Microstreaming and microjetting from collapsing bubbles increase the catalytic rate by favouring a high mass-transport rate, improving the adsorption and desorption process of the reactants [6]. As a cavitation bubble inertially collapses, the gas and vapour trapped in the bubble becomes extremely hot and pressurised pyrolyzing molecules into free radicals (sonolysis). The catalytic reaction rate may be increased at these higher temperatures due to the reduction in reaction activation energy and improved electron transfer rate [42]. Furthermore, sonoluminescence from bubble collapse may also occur and mediate photoactivation of AuNCs [43], [44]. This photophysical response of conduction electrons in metal nanoparticles to incident photons induces a collective coherent oscillation of free electrons (conduction band electrons) in AuNCs that enhances catalytic activity [45]. For the reactions studied here, 4-NP may have been hydrogenated to form 4-aminophenol by way of mechanical-, thermal-, and photo-activation, or have been attacked by hydroxyl radicals to generate organic radicals or some other intermediates. Similarly, electrons and hydroxyl radicals were likely the main species to decolour the MB solution. Electrons may convert MB to colourless leucomethylene blue. Free radical species may react with the MB cation break down the molecule and form a wide range of degradation intermediates that may be further decomposed and mineralized into CO_2_, H_2_O, SO_4_^2^^-^ and NO_3_- [46]. Our approach demonstrated that control of cavitation at catalytic sites by structuring the catalyst to trap gas (gs-AuNCs) provided rapid degradation of pollutants. Though only 4-NP and MB were studied in this report as a proof-of-concept, it is important to emphasize that this method to couple cavitation events and catalysts may be a simple strategy to improve the efficacy of metal catalysts for other advanced catalytic processes [47].

## 4 Conclusion

In summary, we manufactured ultrasound-responsive gold nanocones for efficient, green sonochemical processing. AuNCs were investigated for their potential to nucleate cavitation and enhance sonocatalytic degradation of both MB and 4-nitrophenol. Our results indicated that the gas trapping cavity of the Au catalyst enabled more effective catalytic sonochemistry. By trapping gas onto the AuNCs, the particles nucleated inertial cavitation at the site of the catalyst at relatively low acoustic pressures (1.1 MHz focussed ultrasound, 5.0 MPa peak negative pressure) change the local physicochemical environment to create a high-energy microreactor primed for the catalytic reactions. This resulted in catalytic reaction rates at least one order of magnitude faster than existing literature using comparable Au sizes. Furthermore, the cavitation energy from gs-AuNCs indicated a direct positive correlation to chemical degradation, validating the importance of cavitation events and colocalization of the events to photocatalytic sites. The submicron size of the particles further allows us to filter the catalysts by centrifugation to recycle and reuse for continued ultrasound exposure with negligible changes to sonoreactivity. In short, we showed that simple structuring of the shape of Au catalysts will greatly improve the catalyst reaction rates. The idea of using catalysts as cavitation nuclei to enhance the reactivity of the catalytic agents is not limited to the examples provided in this report, and therefore suggests that this approach may be a simple strategy to improve the efficacy of other catalytic sonochemical reactions.

## Funding sources

Funding was provided by NTU start-up grant (M4081814.120).

## Declaration of Competing Interest

The authors declare that they have no known competing financial interests or personal relationships that could have appeared to influence the work reported in this paper.

## References

[1] Green I.X., Tang W., Neurock M., Yates J.T. (2011). Spectroscopic observation of dual catalytic sites during oxidation of CO on a Au/TiO(2) catalyst. Science.

[2] Zhu W., Liu L., Yue Z., Zhang W., Yue X., Wang J., Yu S., Wang L., Wang J. (2017). Au Promoted Nickel-Iron Layered Double Hydroxide Nanoarrays: A Modular Catalyst Enabling High-Performance Oxygen Evolution. ACS Appl. Mater. Interfaces.

[3] Schubert M.M., Hackenberg S., van Veen A.C., Muhler M., Plzak V., Behm R.J. (2001). CO Oxidation over Supported Gold Catalysts—“Inert” and “Active” Support Materials and Their Role for the Oxygen Supply during Reaction. J. Catal..

[4] Ishida T., Murayama T., Taketoshi A., Haruta M. (2020). Importance of Size and Contact Structure of Gold Nanoparticles for the Genesis of Unique Catalytic Processes. Chem. Rev..

[5] Argyle M.D., Bartholomew C.H. (2015). Heterogeneous catalyst deactivation and regeneration: A review. Catalysts.

[6] Gu S., Wunder S., Lu Y., Ballauff M., Fenger R., Rademann K., Jaquet B., Zaccone A. (2014). Kinetic Analysis of the Catalytic Reduction of 4-Nitrophenol by Metallic Nanoparticles. J. Phys. Chem. C.

[7] Panigrahi S., Basu S., Praharaj S., Pande S., Jana S., Pal A., Ghosh S.K., Pal T. (2007). Synthesis and Size-Selective Catalysis by Supported Gold Nanoparticles: Study on Heterogeneous and Homogeneous Catalytic Process. J. Phys. Chem. C.

[8] Sajjadi S., Khataee A., Kamali M. (2017). Sonocatalytic degradation of methylene blue by a novel graphene quantum dots anchored CdSe nanocatalyst. Ultrason. Sonochem..

[9] Suslick K.S. (1990). Sonochemistry. Science.

[10] Suslick K.S. (1991). The sonochemical hot spot. The Journal of the Acoustical Society of America.

[11] Rekhviashvili S.S. (2008). Single-bubble sonoluminescence model. Tech. Phys. Lett..

[12] Lin X., Liu S., Zhang X., Zhu R., Chen S., Chen X., Song J., Yang H. (2020). An Ultrasound Activated Vesicle of Janus Au-MnO Nanoparticles for Promoted Tumor Penetration and Sono-Chemodynamic Therapy of Orthotopic Liver Cancer. Angew. Chem. Int. Ed. Engl..

[13] BlakePerutz J.R., Suslick K.S., Didenko Y., Fang M.M., Hyeon T., Kolbeck K.J., McNamara W.B., Mdleleni M.M., Wong M. (1999). Acoustic cavitation and its chemical consequences, Philosophical Transactions of the Royal Society of London. Series A: Mathematical, Physical and Engineering Sciences.

[14] Briggs H.B., Johnson J.B., Mason W.P. (1947). Properties of Liquids at High Sound Pressure. J. Acoust. Soc. Am..

[15] Kwan J.J., Graham S., Myers R., Carlisle R., Stride E., Coussios C.C. (2015). Ultrasound-induced inertial cavitation from gas-stabilizing nanoparticles. Phys. Rev. E: Stat. Nonlinear Soft Matter Phys..

[16] Mannaris C., Teo B.M., Seth A., Bau L., Coussios C., Stride E. (2018). Gas-Stabilizing Gold Nanocones for Acoustically Mediated Drug Delivery. Adv Healthc Mater.

[17] Beguin E., Shrivastava S., Dezhkunov N.V., McHale A.P., Callan J.F., Stride E. (2019). Direct Evidence of Multibubble Sonoluminescence Using Therapeutic Ultrasound and Microbubbles. ACS Appl. Mater. Interfaces.

[18] Jonnalagadda U.S., Su X., Kwan J.J. (2021). Nanostructured TiO2 cavitation agents for dual-modal sonophotocatalysis with pulsed ultrasound. Ultrason. Sonochem..

[19] Xu Y., Shi X., Hua R., Zhang R., Yao Y., Zhao B.o., Liu T., Zheng J., Lu G. (2020). Remarkably catalytic activity in reduction of 4-nitrophenol and methylene blue by Fe3O4@COF supported noble metal nanoparticles. Appl. Catal. B.

[20] Yao T., Cui T., Wang H., Xu L., Cui F., Wu J. (2014). A simple way to prepare Au@polypyrrole/Fe3O4 hollow capsules with high stability and their application in catalytic reduction of methylene blue dye. Nanoscale.

[21] Fu Y., Huang T., Jia B., Zhu J., Wang X. (2017). Reduction of nitrophenols to aminophenols under concerted catalysis by Au/g-C3N4 contact system. Appl. Catal. B.

[22] Evangelista V., Acosta B., Miridonov S., Smolentseva E., Fuentes S., Simakov A. (2015). Highly active Au-CeO2@ZrO2 yolk–shell nanoreactors for the reduction of 4-nitrophenol to 4-aminophenol. Appl. Catal. B.

[23] Zhang P., He J., Ma X., Gong J., Nie Z. (2013). Ultrasound assisted interfacial synthesis of gold nanocones. Chem. Commun. (Camb.).

[24] Kwan J.J., Myers R., Coviello C.M., Graham S.M., Shah A.R., Stride E., Carlisle R.C., Coussios C.C. (2015). Ultrasound-Propelled Nanocups for Drug Delivery. Small.

[25] Su X., Rakshit M., Das P., Gupta I., Das D., Pramanik M., Ng K.W., Kwan J. (2021). Ultrasonic Implantation and Imaging of Sound-Sensitive Theranostic Agents for the Treatment of Arterial Inflammation. ACS Appl. Mater. Interfaces.

[26] Graham S.M., Carlisle R., Choi J.J., Stevenson M., Shah A.R., Myers R.S., Fisher K., Peregrino M.B., Seymour L., Coussios C.C. (2014). Inertial cavitation to non-invasively trigger and monitor intratumoral release of drug from intravenously delivered liposomes. J. Control. Release.

[27] Su X., Thomas R.G., Bharatula L.D., Kwan J.J. (2019). Remote targeted implantation of sound-sensitive biodegradable multi-cavity microparticles with focused ultrasound. Sci. Rep..

[28] Borkent B.M., Gekle S., Prosperetti A., Lohse D. (2009). Nucleation threshold and deactivation mechanisms of nanoscopic cavitation nuclei. Phys. Fluids.

[29] Midathana V.R., Moholkar V.S. (2009). Mechanistic Studies in Ultrasound-Assisted Adsorption for Removal of Aromatic Pollutants. Ind. Eng. Chem. Res..

[30] McKenzie T.G., Karimi F., Ashokkumar M., Qiao G.G. (2019). Ultrasound and Sonochemistry for Radical Polymerization: Sound Synthesis. Chemistry.

[31] Paris J.L., Mannaris C., Cabañas M.V., Carlisle R., Manzano M., Vallet-Regí M., Coussios C.C. (2018). Ultrasound-mediated cavitation-enhanced extravasation of mesoporous silica nanoparticles for controlled-release drug delivery. Chem. Eng. J..

[32] Chappell M.A., Payne S.J. (2007). The effect of cavity geometry on the nucleation of bubbles from cavities. J. Acoust. Soc. Am..

[33] Yildirim A., Chattaraj R., Blum N.T., Goldscheitter G.M., Goodwin A.P. (2016). Stable Encapsulation of Air in Mesoporous Silica Nanoparticles: Fluorocarbon-Free Nanoscale Ultrasound Contrast Agents. Adv Healthc Mater.

[34] Rooze J., Rebrov E.V., Schouten J.C., Keurentjes J.T. (2013). Dissolved gas and ultrasonic cavitation–a review. Ultrason. Sonochem..

[35] Wood R.J., Lee J., Bussemaker M.J. (2017). A parametric review of sonochemistry: Control and augmentation of sonochemical activity in aqueous solutions. Ultrason. Sonochem..

[36] Thawarkar S.R., Thombare B., Munde B.S., Khupse N.D. (2018). Kinetic investigation for the catalytic reduction of nitrophenol using ionic liquid stabilized gold nanoparticles. RSC Adv..

[37] Zhou J., Duan B.o., Fang Z., Song J., Wang C., Messersmith P.B., Duan H. (2014). Interfacial assembly of mussel-inspired au@ag@ polydopamine core-shell nanoparticles for recyclable nanocatalysts. Adv. Mater..

[38] Ilgin P., Ozay O., Ozay H. (2019). A novel hydrogel containing thioether group as selective support material for preparation of gold nanoparticles: Synthesis and catalytic applications. Appl. Catal. B.

[39] Ji Z., Shen X., Xu Y., Zhu G., Chen K. (2014). Anchoring noble metal nanoparticles on CeO2 modified reduced graphene oxide nanosheets and their enhanced catalytic properties. J. Colloid Interface Sci..

[40] Wei J., Wang H., Deng Y., Sun Z., Shi L., Tu B.o., Luqman M., Zhao D. (2011). Solvent evaporation induced aggregating assembly approach to three-dimensional ordered mesoporous silica with ultralarge accessible mesopores. J. Am. Chem. Soc..

[41] Kwan J.J., Lajoinie G., de Jong N., Stride E., Versluis M., Coussios C.C. (2016). Ultrahigh-Speed Dynamics of Micrometer-Scale Inertial Cavitation from Nanoparticles. Phys. Rev. Appl.

[42] Pollet B.G. (2019). Does power ultrasound affect heterogeneous electron transfer kinetics?. Ultrason. Sonochem..

[43] Kumari G.V., JothiRajan M.A., Mathavan T. (2018). Pectin functionalized gold nanoparticles towards singlet oxygen generation. Mater. Res. Express.

[44] Amendola V., Pilot R., Frasconi M., Maragò O.M., Iatì M.A. (2017). Surface plasmon resonance in gold nanoparticles: a review. J. Phys.: Condens. Matter.

[45] Gao S., Zhang Z., Liu K., Dong B. (2016). Direct evidence of plasmonic enhancement on catalytic reduction of 4-nitrophenol over silver nanoparticles supported on flexible fibrous networks. Appl. Catal. B.

[46] Ma C., Feng S., Zhou J., Chen R., Wei Y.u., Liu H., Wang S. (2019). Enhancement of H2O2 decomposition efficiency by the co-catalytic effect of iron phosphide on the Fenton reaction for the degradation of methylene blue. Appl. Catal. B.

[47] Wang J., Guo Y., Liu B., Jin X., Liu L., Xu R., Kong Y., Wang B. (2011). Detection and analysis of reactive oxygen species (ROS) generated by nano-sized TiO2 powder under ultrasonic irradiation and application in sonocatalytic degradation of organic dyes. Ultrason. Sonochem..

