# Supplementary material for: Unsupported gold nanocones as sonocatalytic agents with enhanced catalytic properties
Source: Ultrason Sonochem. 2021 Sep 13;79:105753. doi: 10.1016/j.ultsonch.2021.105753 (PMC8473759; doi:10.1016/j.ultsonch.2021.105753)
Supplement: Supplementary data 1 [file mmc1.docx]

Supplementary Information for

Unsupported Gold Nanocones as Sonocatalytic Agents with Enhanced Catalytic Properties

Xiaoqian Su,^1^ Umesh Sai Jonnalagadda,^1^ Lakshmi Deepika Bharatula,^1^ James Jing Kwan^2^*

1. School of Chemical and Biomedical Engineering, Nanyang Technological University, 62 Nanyang Drive, Singapore 637459
2. Department of Engineering Sciences, University of Oxford, Oxford, United Kingdom OX1 3PJ

* Corresponding author email: [james.kwan@eng.ox.ac.uk](mailto:james.kwan@eng.ox.ac.uk)

This supplement contains:

Supplementary Figures S1—7

Supplementary Table S1

**Supplementary Figures:**


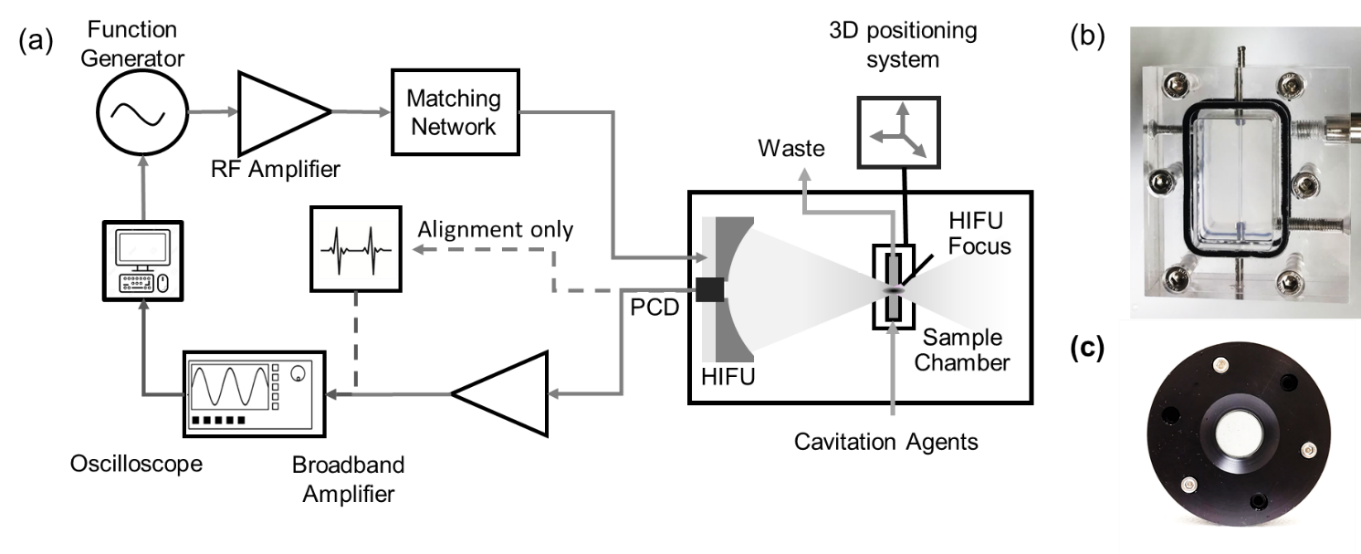


**Figure S1**. Acoustic setup. (a) Schematic of the ultrasound setup. (b) Continues flow sample chamber. (c) static flow sample chamber. The acoustically transparent agarose continuous flow sample chamber was made from a 2% (w/v) of agarose solution, which was boiled and degassed for 30 min to prevent cavitation as a result of endogenous bubbles. The agarose solution was then poured into a bespoke cuboid mould (50 mm in length × 30 mm in width) and sealed with acoustically transparent windows. A 1.6 mm steel rod was threaded through the mould. After gelation was completed, the rod was removed, creating a flow channel. The static chamber consists of an acoustically transparent window with a diameter of 18 mm and a depth of 5 mm (volume 1 mL).


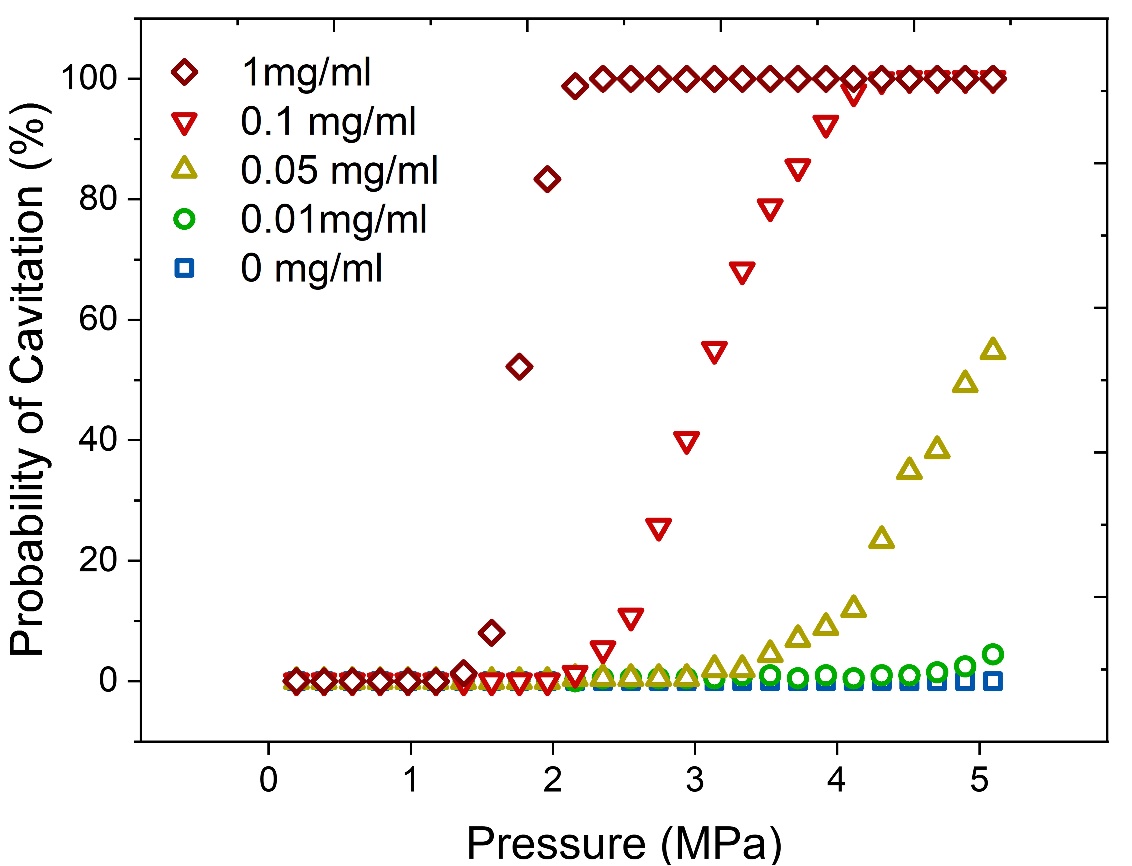


**Figure S2**. Probability of Cavitation of gs-AuNCs at a concentration of 0, 0.01, 0.05, 0.1 and 1 mg/mL exposed to 20 cycle bursts of 1.1 MHz ultrasound for 20 s with increasing peak negative pressure amplitude from 0.2-5.2 MPa peak negative pressure at a pulse repetition time of 0.1 s.


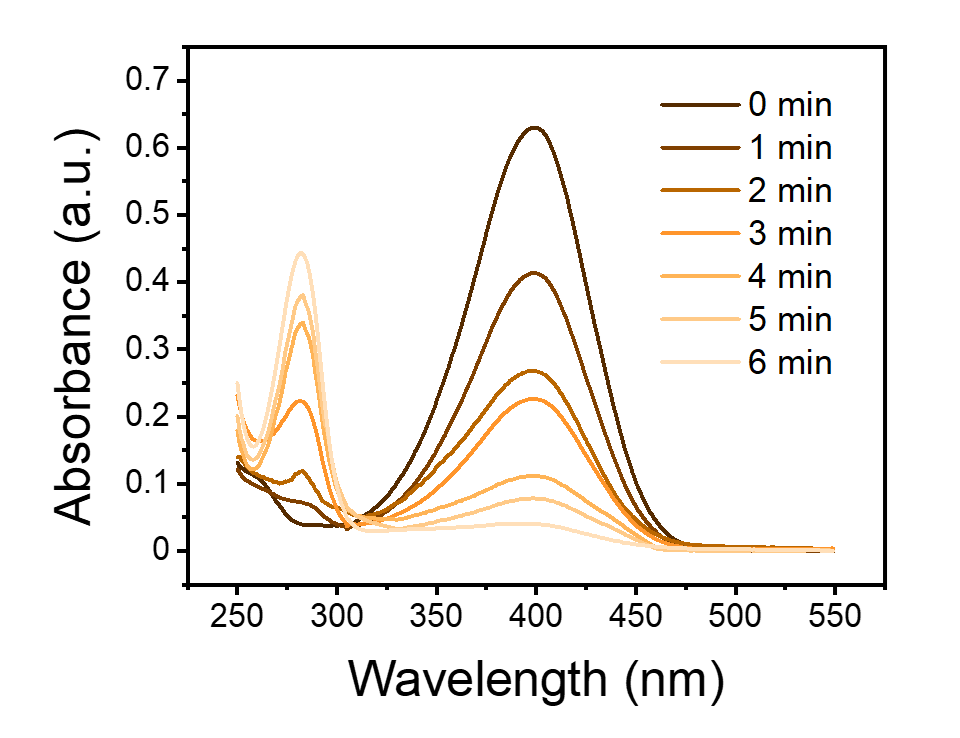


**Figure S3.** UV-vis spectral changes during the sonocatalytic degradation of 4-nitrophenol against the reaction time.

**
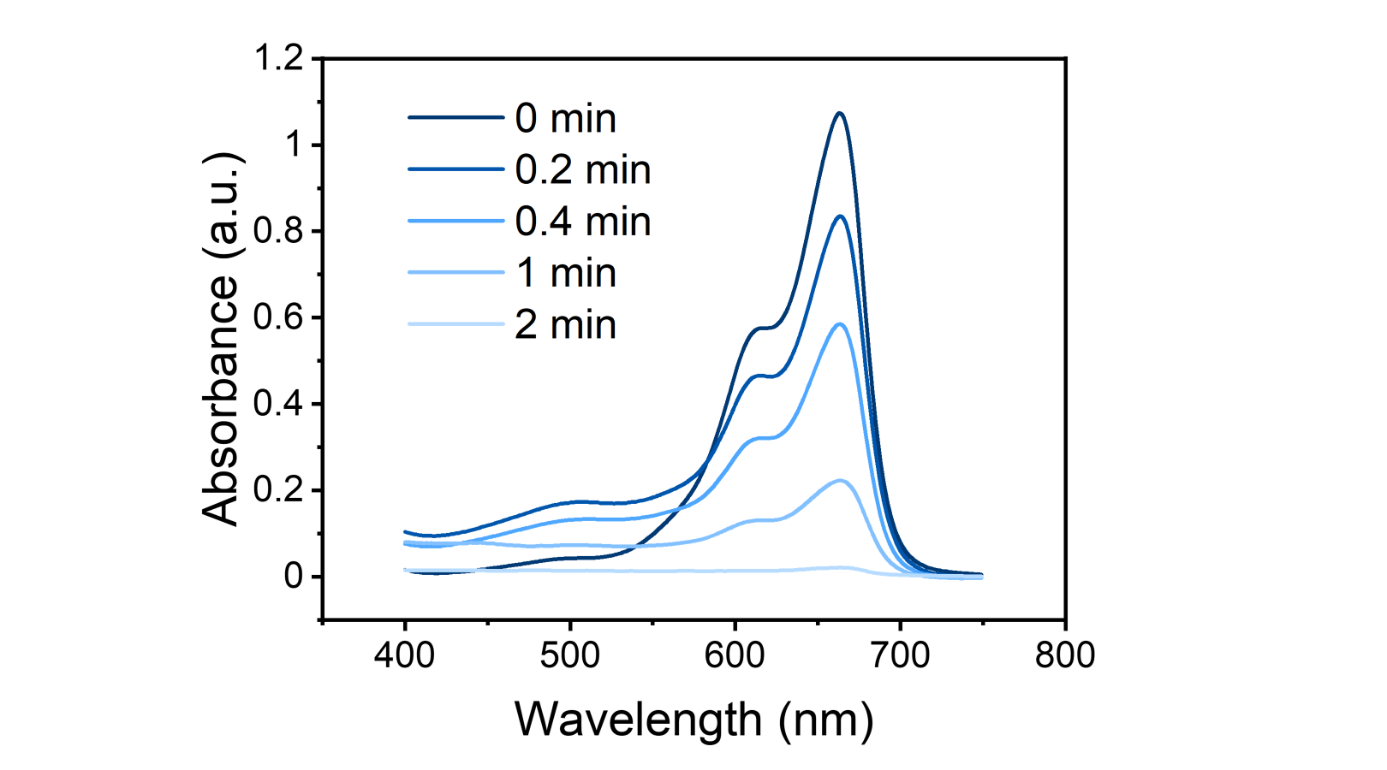
**

**Figure S4.** UV-vis spectral changes during the sonocatalytic degradation of MB against the reaction time.


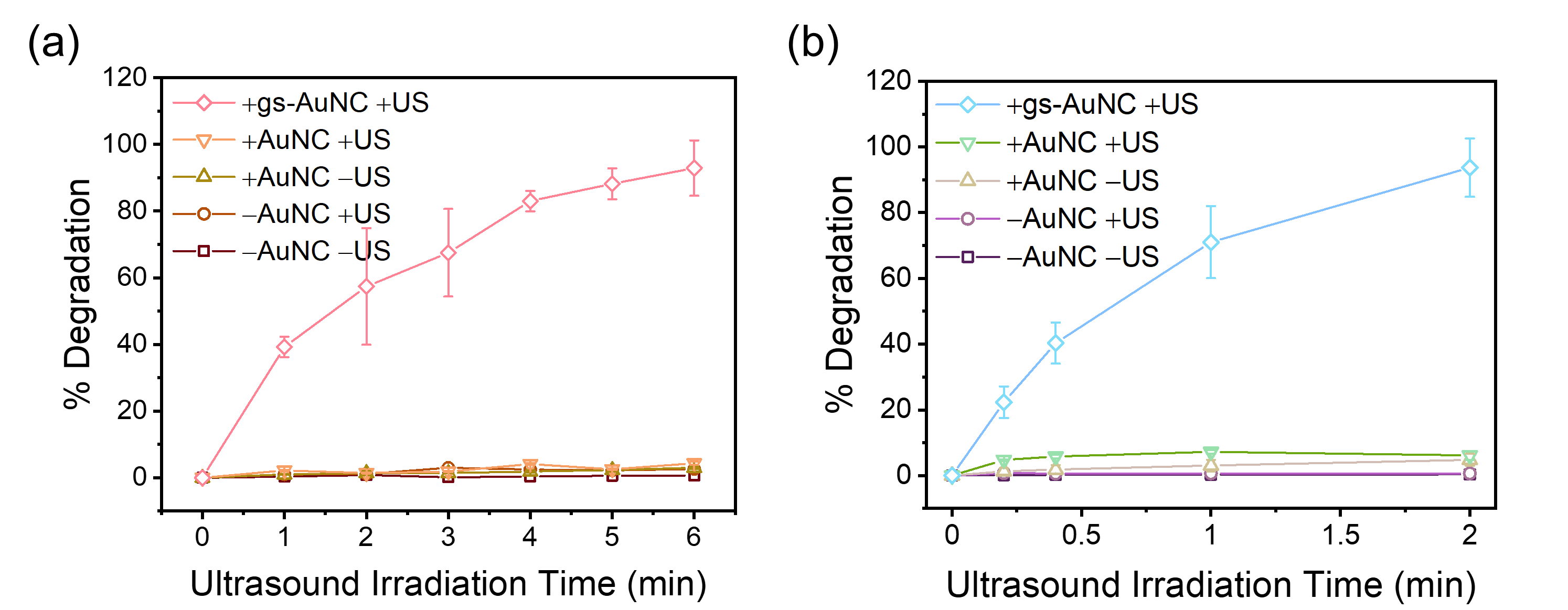


**Figure S5.** (a) Sonocatalytic degradation of 4-NP (a) and MB (b) at different conditions. Data represented as mean ± SD (n=3).

**
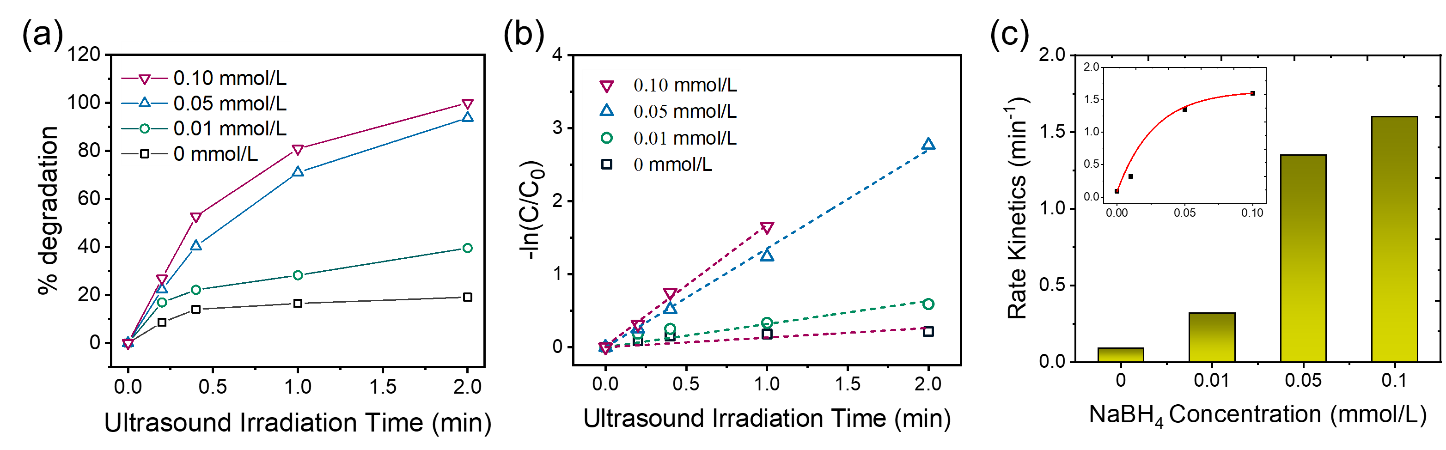
**

**Figure S6.** The effect of the NaBH_4_ dosage on sonocatalytic degradation of MB**.**

**
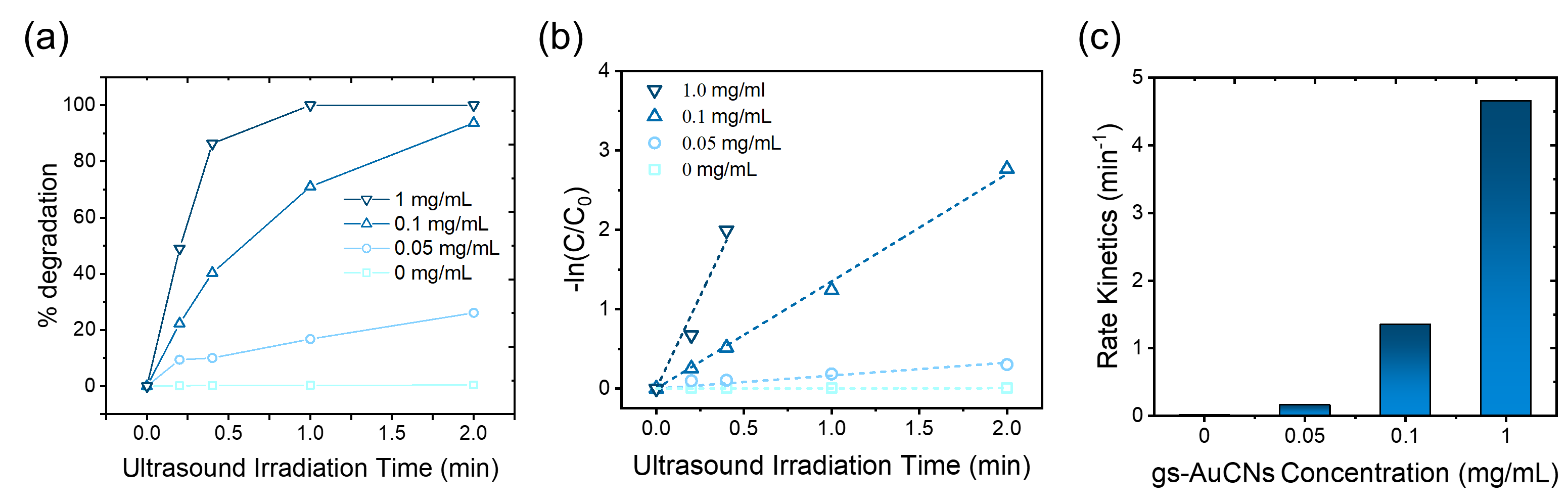
**

**Figure S7.** The effect of the gs-AuNCs concentration on sonocatalytic degradation of MB.

**Table S1.** Comparison of catalytic activity of Au by degradation of methylene blue

| **Catalysts** | Au Size (nm) | Dose (mg·mL⁻¹) | *k*  (min⁻¹) | K^a^ *,* (min⁻¹mg⁻¹) | Ref |
| --- | --- | --- | --- | --- | --- |
| Au+Ultrasound^b^ | 170 | 0.1 | 1.35 | 13.50 | This work |
| Au | 170 | 0.1 | 0.04 | 0.40 | This work |
| Au/Zeolite | 6 | 5 | 0.07 | 0.012 | 1 |
| Fe_3_O_4_@COF-Au | 4 | 0.27 | 0.9 | 3.333 | 2 |
| Au@polypyrrole/Fe_3_O_4_ | 15 | 0.57 | 0.266 | 0.466 | 3 |
| Au/InBi-3D + 300 W Xe lamp | 4 | 0.08 | 0.236 | 2.95 | 4 |

^a^ Normalised rate constant K is the ratio of the apparent rate constant (k) to the mass of catalyst (mg).

^b^ 1.1 MHz focussed ultrasound irradiation at 5.0 MPa peak negative pressure and 20% duty cycle.

**References**

1. León, E. R.; Rodríguez, E. L.; Beas, C. R.; Plascencia-Villa, G.; Palomares, R. A. I. Study of methylene blue degradation by gold nanoparticles synthesized within natural zeolites, *J. Nanomater.* 2016 (2016).

2. Xu, Y.; Shi, X.; Hua, R.; Zhang, R.; Yao, Y.; Zhao, B.; Liu, T.; Zheng, J.; Lu, G. Remarkably catalytic activity in reduction of 4-nitrophenol and methylene blue by Fe_3_O_4_@COF supported noble metal nanoparticles. *Appl. Catal. B.* 2020, 260, 118-142.

3. Yao, T.; Cui, T.; Wang, H.; Xu, L.; Cui, F.; Wu, J. A simple way to prepare Au@polypyrrole/Fe_3_O_4_ hollow capsules with high stability and their application in catalytic reduction of methylene blue dye. *Nanoscal*e. 2014, 6, 7666–7674.

4. Deng, J. K.; Zang, J.; Han, H.; Arandiyan, J.; Dai, H. Fabrication and high photocatalytic performance of noble metal nanoparticles supported on 3DOM InVO_4_–BiVO_4_ for the visible-light-driven degradation of rhodamine B and methylene blue. *Appl. Catal. B.* 2015, 165, 285–295.
